# Supplementary material for: Impact of body weight gain on hepatic metabolism and hepatic inflammatory cytokines in comparison of Shetland pony geldings and Warmblood horse geldings
Source: PeerJ. 2019 Jun 7;7:e7069. doi: 10.7717/peerj.7069 (PMC6557249; doi:10.7717/peerj.7069)
Supplement: Supplemental Information 1 — 1Mean of two independent evaluators. Age, BW, leukocytes, erythrocytes and hemoglobin are presented as mean (± SD); BCS, CNS and ACTH are presented as median (25th/75th percentiles). [file peerj-07-7069-s001.docx]

Descriptive data of the ponies and horses at the beginning of the study

|  | | Shetland ponies | Warmblood horses |
| --- | --- | --- | --- |
| Sex | | 10 geldings | 9 geldings |
| Age, years | | 6 ± 3 | 10 ± 3 |
| Body weight, kg | | 118 ± 29 | 589 ± 58 |
| Body condition score (BCS),  0-5^1^ | | 2.3 (1.2/3.4) | 2.7 (2.1/3.2) |
| Cresty neck score (CNS),  0-5^2^ | | 2.5 (0.8/3) | 2 (1.8/2.3) |
| Plasma adrenocorticotropic hormone (ACTH), pg/mL  Threshold: 50 pg/mL | | 15.9 (12.8/17.1) | 16.4 (15.1/19.6) |
| Leukocytes (G/L) | t0  t2  t5 | 7.87 ± 1.26  6.18 ± 2.23  6.93 ± 2.09 | 5.14 ± 0.72  5.58 ± 1.6  5.88 ± 1.12 |
| Erythrocytes (T/L) | t0  t2  t5 | 6.15 ± 0.6  6.53 ± 0.59  6.87 ± 0.96 | 6.52 ± 0.43  7.39 ± 0.8  7.92 ± 0.98 |
| Hemoglobin (mmol/L) | t0  t2  t5 | 6.17 ± 0.67  6.58 ± 0.61  6.93 ± 0.77 | 6.93 ± 0.43  7.71 ± 0.72  8.13 ± 0.69 |

^1^Mean of two independent evaluators. Age, BW, leukocytes, erythrocytes and hemoglobin are presented as mean (± SD); BCS, CNS and ACTH are presented as median (25^th^/75^th^ percentiles).
