# Supplementary material for: Impact of body weight gain on hepatic metabolism and hepatic inflammatory cytokines in comparison of Shetland pony geldings and Warmblood horse geldings
Source: PeerJ. 2019 Jun 7;7:e7069. doi: 10.7717/peerj.7069 (PMC6557249; doi:10.7717/peerj.7069)
Supplement: Supplemental Information 2 — Data are expressed as mean ± SD. [file peerj-07-7069-s002.docx]

Plasma glucose concentrations (mmol/L) and serum insulin concentrations (µU/mL) for two sampling points (0 and 45 minutes after insulin bolus) during combined glucose insulin test (CGIT) at basal measurements (t0), after one year (t2) and after two years (t5) of excess energy intake.

| Variable | Sampling point | Breed | t0 | | t2 | | t5 | |
| --- | --- | --- | --- | --- | --- | --- | --- | --- |
| Glucose  (mmol/L) | 0 | Ponies | 3.53 | ± 0.63 | 3.93 | ± 0.38 | 4.34 | ± 0.86 |
|  |  | Horses | 4.08 | ± 0.20 | 4.52 | ± 0.22 | 4.41 | ± 0.47 |
|  | 45 | Ponies | 3.79 | ± 1.24 | 2.4 | ± 1.08 | 3.25 | ± 1.59 |
|  |  | Horses | 3.72 | ± 1.34 | 3.18 | ± 0.98 | 3.89 | ± 1.94 |
| Insulin (µU/mL) | 0 | Ponies | 4.26 | ± 1.36 | 7.93 | ± 5.75 | 13.9 | ± 14.9 |
|  |  | Horses | 6.32 | ± 2.35 | 9.3 | ± 3.18 | 15.1 | ± 10.3 |
|  | 45 | Ponies | 26.1 | ± 13.1 | 37.6 | ± 11. 1 | 62.2 | ± 38.4 |
|  |  | Horses | 37.5 | ± 14.1 | 51 | ± 16.2 | 85.6 | ± 54.4 |

Data are expressed as mean ± SD.
