# Supplementary material for: Impact of body weight gain on hepatic metabolism and hepatic inflammatory cytokines in comparison of Shetland pony geldings and Warmblood horse geldings
Source: PeerJ. 2019 Jun 7;7:e7069. doi: 10.7717/peerj.7069 (PMC6557249; doi:10.7717/peerj.7069)
Supplement: Supplemental Information 3 — Data are expressed as mean ± SD.. [file peerj-07-7069-s003.docx]

Serum amyloid A (SAA) concentrations (µg/mL) at basal measurements (t0), after one year (t2) and after two years (t5) of excess energy intake in ponies and horses.

| Variable | Breed | t0 | t2 | t5 |
| --- | --- | --- | --- | --- |
| SAA  (µg/mL) | Ponies | 0.35 ± 0.5 | 0.24 ± 0.22 | 0.1 ± 0 |
|  | Horses | 0.1 ± 0 | 0.1 ± 0 | 0.1 ± 0 |

Data are expressed as mean ± SD.
