# Supplementary material for: Impact of body weight gain on hepatic metabolism and hepatic inflammatory cytokines in comparison of Shetland pony geldings and Warmblood horse geldings
Source: PeerJ. 2019 Jun 7;7:e7069. doi: 10.7717/peerj.7069 (PMC6557249; doi:10.7717/peerj.7069)
Supplement: Supplemental Information 4 — Data are presented as medians and 25th/75th percentiles.. [file peerj-07-7069-s004.docx]

Hepatic mRNA levels of TNFα, IL-6, FABP1 and CD68 (x-fold) at basal measurements (t0), after one year (t2) and after two years (t5) of excess energy intake in ponies and horses.

| Variable | Breed | t0 | t2 | t5 |
| --- | --- | --- | --- | --- |
| TNFα  (x-fold) | Ponies | 0.62 (0.46/0.81) | 0.69 (0.45/0.79) | 0.73 (0.67/1.18) |
|  | Horses | 0.45 (0.37/0.51) | 0.52 (0.33/0.71) | 0.64 (0.49/0.77) |
| IL-6  (x-fold) | Ponies | 0.29  (0.25/0.8) | 0.27 (0.24/0.34) | 0.62  (0.4/0.79) |
|  | Horses | 0.29 (0.25/0.33) | 0.27 (0.17/0.38) | 0.44 (0.26/0.53) |
| FABP1  (x-fold) | Ponies | 0.71 (0.55/0.73) | 0.4  (0.36/0.6) | 0.68 (0.42/0.77) |
|  | Horses | 0.71 (0.42/0.81) | 0.52 (0.49/0.64) | 0.71  (0.5/0.94) |
| CD68  (x-fold) | Ponies | 0.72 (0.65/0.83) | 0.77 (0.48/1.15) | 0.65 (0.52/0.76) |
|  | Horses | 0.53 (0.43/0.64) | 0.53 (0.47/0.55) | 0.49  (0.4/0.52) |

data are presented as medians and 25th/75th percentiles
